# Correction: Quality of life in adults with Down syndrome: A mixed methods systematic review

**DOI:** 10.1371/journal.pone.0315110

**Published:** 2024-12-03

**Authors:** Ogochukwu Ann Ijezie, Jane Healy, Philip Davies, Emili Balaguer-Ballester, Vanessa Heaslip

The number of duplicate records removed is incorrect in Fig 1. It should be 570 instead of 896. Please see the correct Fig 1 here.

**Fig 1:**
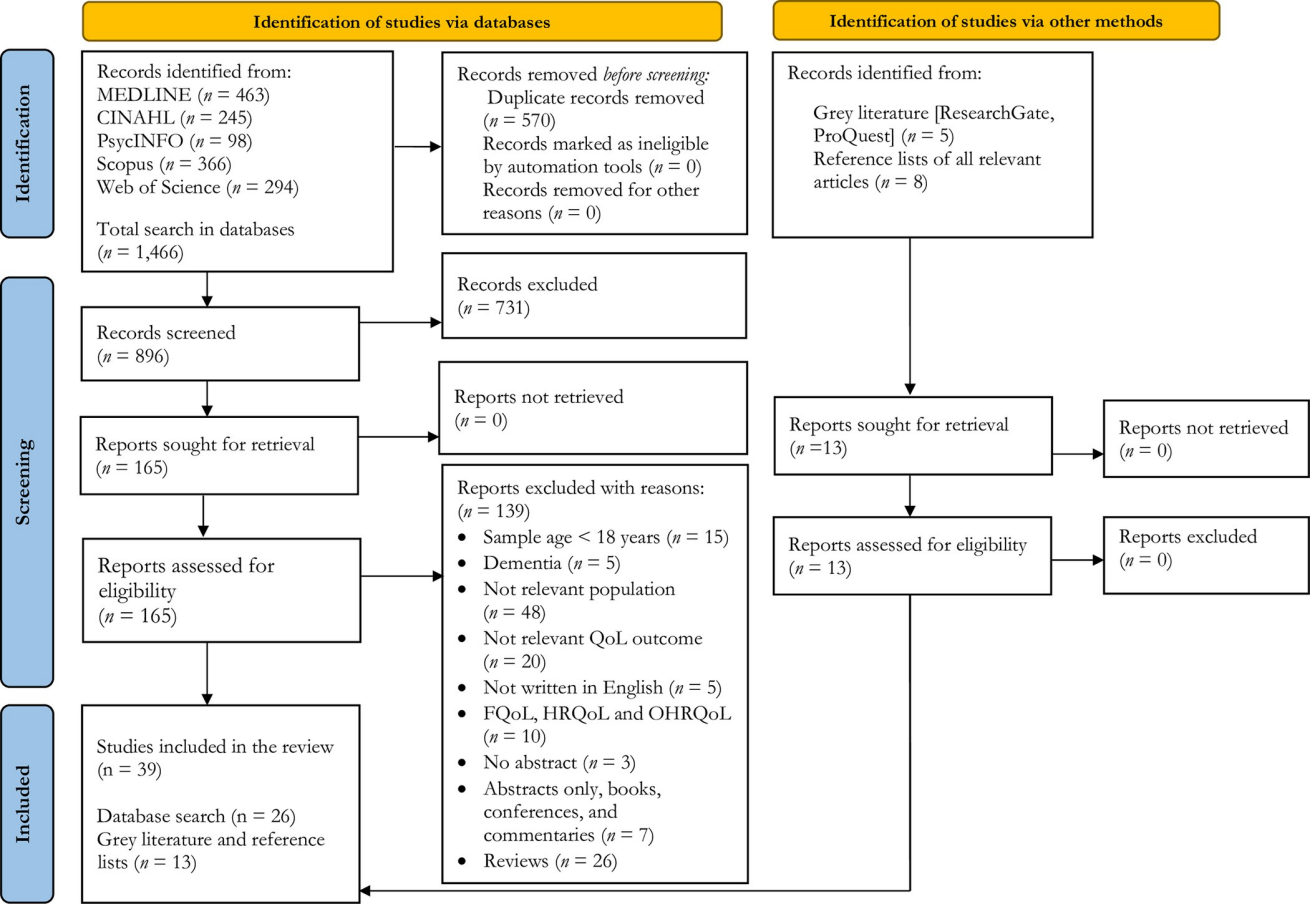
PRISMA flowchart of the study selection and inclusion process. Note: QoL = Quality of life; OHRQoL = Oral health-related quality of life.
