# Supplementary material for: Quality of life in adults with Down syndrome: A mixed methods systematic review
Source: PLoS One. 2023 May 1;18(5):e0280014. doi: 10.1371/journal.pone.0280014 (PMC10150991; doi:10.1371/journal.pone.0280014)
Supplement: S1 Table — (DOCX) [file pone.0280014.s002.docx]

**S1 Table.** **Search strategy**

| **Electronic databases** | **Search** | **Query (through EBSCO interface)** | **Records retrieved** |
| --- | --- | --- | --- |
| **MEDLINE** | #1 | (MH "Down syndrome") OR TIAB ("Down* syndrome" OR "Trisomy 21") | 32,324 |
|  | #2 | (MH "Intellectual Disability+") OR (MH "Learning Disabilities+") OR (MH "Disabled Persons+") OR (MH "Developmental Disabilities") OR TIAB ("Intellectual and Developmental Disabilit*" OR "Intellectual Disabilit*" OR "Developmental Disabilit*" OR "Learning Disabilit*" OR "Disabled Person*" OR "Disabled People" OR "Disabled Individual*" OR "Learning Disorder*" OR "Disabilit*" OR "People with Intellectual and Developmental Disabilit*" OR "People with Intellectual Disabilit*" OR "People with Developmental Disabilit*" OR "People with Learning Disabilit*" OR "People with Learning Disorder*" OR "People with Disabilit*" OR "Person* with Intellectual and Developmental Disabilit*" OR "Person* with Intellectual Disabilit*" OR "Person* with Developmental Disabilit*" OR "Person* with Learning Disabilit*" OR "Person* with Learning Disorder*" OR "Person* with Disabilit*" OR "Individual* with Intellectual and Developmental Disabilit*" OR " Individual* with Intellectual Disabilit*" OR " Individual* with Developmental Disabilit*" OR " Individual* with Learning Disabilit*" OR " Individual* with Learning Disorder*" OR " Individual* with Disabilit*") | 347,162 |
|  | #3 | (MH "Quality of Life") OR (MH "Value of Life") OR TIAB ("Quality of life" OR "QoL" OR "QOL" OR "Well-being" OR "Well being" OR "Wellbeing" OR "Life quality" OR "Quality life" OR "Value of life" OR "Life satisfaction" OR "Quality of living" OR "Standard of living") | 443,211 |
|  | **#4** | **#1 AND #2 AND #3** **AND Source Types: Academic Journals** | **463** |
| **CINAHL** | #1 | (MH "Down syndrome") OR TIAB ("Down* syndrome" OR "Trisomy 21") | 9,311 |
|  | #2 | (MH "Intellectual Disability+") OR (MH "Developmental Disabilities") OR  (MH "Learning Disorders+") OR (MH "Developmental Disabilities") OR TIAB ("Intellectual and Developmental Disabilit*" OR "Intellectual Disabilit*" OR "Developmental Disabilit*" OR "Learning Disabilit*" OR "Disabled Person*" OR "Disabled People" OR "Disabled Individual*" OR "Learning Disorder*" OR "Disabilit*" OR "People with Intellectual and Developmental Disabilit*" OR "People with Intellectual Disabilit*" OR "People with Developmental Disabilit*" OR "People with Learning Disabilit*" OR "People with Learning Disorder*" OR "People with Disabilit*" OR "Person* with Intellectual and Developmental Disabilit*" OR "Person* with Intellectual Disabilit*" OR "Person* with Developmental Disabilit*" OR "Person* with Learning Disabilit*" OR "Person* with Learning Disorder*" OR "Person* with Disabilit*" OR "Individual* with Intellectual and Developmental Disabilit*" OR " Individual* with Intellectual Disabilit*" OR " Individual* with Developmental Disabilit*" OR " Individual* with Learning Disabilit*" OR " Individual* with Learning Disorder*" OR " Individual* with Disabilit*") | 145,155 |
|  | #3 | (MH "Quality of Life") OR TIAB ("Quality of life" OR "QoL" OR "QOL" OR "Well-being" OR "Well being" OR "Wellbeing" OR "Life quality" OR "Quality life" OR "Value of life" OR "Life satisfaction" OR "Quality of living" OR "Standard of living") | 233,463 |
|  | **#4** | **#1 AND #2 AND #3** **AND Source Types: Academic Journals** | **245** |
| **PsycINFO** | #1 | TIAB ("Down* syndrome" OR "Trisomy 21") | 7,604 |
|  | #2 | TIAB ("Intellectual and Developmental Disabilit*" OR "Intellectual Disabilit*" OR "Developmental Disabilit*" OR "Learning Disabilit*" OR "Disabled Person*" OR "Disabled People" OR "Disabled Individual*" OR "Learning Disorder*" OR "Disabilit*" OR "People with Intellectual and Developmental Disabilit*" OR "People with Intellectual Disabilit*" OR "People with Developmental Disabilit*" OR "People with Learning Disabilit*" OR "People with Learning Disorder*" OR "People with Disabilit*" OR "Person* with Intellectual and Developmental Disabilit*" OR "Person* with Intellectual Disabilit*" OR "Person* with Developmental Disabilit*" OR "Person* with Learning Disabilit*" OR "Person* with Learning Disorder*" OR "Person* with Disabilit*" OR "Individual* with Intellectual and Developmental Disabilit*" OR " Individual* with Intellectual Disabilit*" OR " Individual* with Developmental Disabilit*" OR " Individual* with Learning Disabilit*" OR " Individual* with Learning Disorder*" OR " Individual* with Disabilit*") | 122,104 |
|  | #3 | TIAB ("Quality of life" OR "QoL" OR "QOL" OR "Well-being" OR "Well being" OR "Wellbeing" OR "Life quality" OR "Quality life" OR "Value of life" OR "Life satisfaction" OR "Quality of living" OR "Standard of living") | 167,102 |
|  | **#4** | **#1 AND #2 AND #3** **AND Source Types: Academic Journals** | **98** |
| **Scopus** | #1 | TITLE-ABS-KEY ("Down* syndrome" OR "Trisomy 21" ) AND ( LIMIT-TO ( DOCTYPE , "ar" ) OR LIMIT-TO ( DOCTYPE , "re" ) ) | 38,779 |
|  | #2 | TITLE-ABS-KEY ( "Intellectual and Developmental Disabilit*" OR "Intellectual Disabilit*" OR "Developmental Disabilit*" OR "Learning Disabilit*" OR "Disabled Person*" OR "Disabled People" OR "Disabled Individual*" OR "Learning Disorder*" OR "Disabilit*" OR "People with Intellectual and Developmental Disabilit*" OR "People with Intellectual Disabilit*" OR "People with Developmental Disabilit*" OR "People with Learning Disabilit*" OR "People with Learning Disorder*" OR "People with Disabilit*" OR "Person* with Intellectual and Developmental Disabilit*" OR "Person* with Intellectual Disabilit*" OR "Person* with Developmental Disabilit*" OR "Person* with Learning Disabilit*" OR "Person* with Learning Disorder*" OR "Person* with Disabilit*" OR "Individual* with Intellectual and Developmental Disabilit*" OR " Individual* with Intellectual Disabilit*" OR " Individual* with Developmental Disabilit*" OR " Individual* with Learning Disabilit*" OR " Individual* with Learning Disorder*" OR " Individual* with Disabilit*" ) AND ( LIMIT-TO ( DOCTYPE , "ar" ) OR LIMIT-TO ( DOCTYPE , "re" ) ) | 383,426 |
|  | #3 | TITLE-ABS-KEY ("Quality of life" OR "QoL" OR "QOL" OR "Well-being" OR "Well being" OR "Wellbeing" OR "Life quality" OR "Quality life" OR "Value of life" OR "Life satisfaction" OR "Quality of living" OR "Standard of living" ) AND ( LIMIT-TO ( DOCTYPE , "ar" ) OR LIMIT-TO ( DOCTYPE , "re" ) ) | 631,751 |
|  | **#4** | **#1 AND #2 AND #3** | **366** |
| **Web of Science** | #1 | (TS = ("Down* syndrome" OR "Trisomy 21") )  AND DOCUMENT  TYPES: (Article) | 21,855 |
|  | #2 | (TS = ("Intellectual and Developmental Disabilit*" OR "Intellectual Disabilit*" OR "Developmental Disabilit*" OR "Learning Disabilit*" OR "Disabled Person*" OR "Disabled People" OR "Disabled Individual*" OR "Learning Disorder*" OR "Disabilit*" OR "People with Intellectual and Developmental Disabilit*" OR "People with Intellectual Disabilit*" OR "People with Developmental Disabilit*" OR "People with Learning Disabilit*" OR "People with Learning Disorder*" OR "People with Disabilit*" OR "Person* with Intellectual and Developmental Disabilit*" OR "Person* with Intellectual Disabilit*" OR "Person* with Developmental Disabilit*" OR "Person* with Learning Disabilit*" OR "Person* with Learning Disorder*" OR "Person* with Disabilit*" OR "Individual* with Intellectual and Developmental Disabilit*" OR " Individual* with Intellectual Disabilit*" OR " Individual* with Developmental Disabilit*" OR " Individual* with Learning Disabilit*" OR " Individual* with Learning Disorder*" OR " Individual* with Disabilit*") AND DOCUMENT TYPES: (Article) | 219,696 |
|  | #3 | (TS = ("Quality of life" OR "QoL" OR "QOL" OR "Well-being" OR "Well being" OR "Wellbeing" OR "Life quality" OR "Quality life" OR "Value of life" OR "Life satisfaction" OR "Quality of living" OR "Standard of living") AND DOCUMENT TYPES: (Article) | 414,034 |
|  | **#4** | **#1 AND #2 AND #3** | **294** |

A collated list of the search strategy

| **S/N** | **Databases** | **Results** |
| --- | --- | --- |
| 1 | MEDLINE | 463 |
| 2 | CINAHL | 245 |
| 3 | PsycINFO | 98 |
| 4 | Scopus | 366 |
| 5 | Web of Science | 294 |
| **Total** | | **1,466** |
