# Supplementary material for: Quality of life in adults with Down syndrome: A mixed methods systematic review
Source: PLoS One. 2023 May 1;18(5):e0280014. doi: 10.1371/journal.pone.0280014 (PMC10150991; doi:10.1371/journal.pone.0280014)
Supplement: S2 Table — (DOCX) [file pone.0280014.s003.docx]

**S2 Table.** **Summary characteristics of the included studies**

| **Reference and Geographical location** | **Study design and data collection methods** | **Participant characteristics** | **QoL assessment report methods** | **Key findings** |
| --- | --- | --- | --- | --- |
| Ailey et al. (2006) [1]  United States of America | **Study Design:** Quantitative cross-sectional  **Data Collection:**  A descriptive correlational design via interview | **Sampling technique:** NA  **Sample size:** n = 100  **Age:** Mean age was NA (Range: 30-57 years) **Gender:** 49 males and 51 females  **Race/ Ethnicity:**  61 White  29 African American  10 Hispanic  **Living arrangements:**  51 lived in the family home  49 lived out of the family home where 3 lived independently and 46 in sheltered living **Level of ID:** Mild or moderate | Self-reports and proxy reports (graduate students and professionals in nursing, physical and occupational therapy, exercise physiology, psychology, and public health) | 32% of adults with DS had high depression scores on a depression screening measure, the CDI-S, and 4% met the criteria for major depression on the PIMRA-AD. However, this does not indicate a higher prevalence of depression in individuals with DS than individuals with IDD. 40% of adults with DS reported feeling lonely all the time and either wanting more friends or having no friends. |
| Alderson  (2001) [2]  United Kingdom | **Study Design:**  Exploratory Qualitative  **Data Collection:**  Interviews | **Sampling technique:** NA  **Sample size:** n = 5  **Age:** Mean age = 27.6 years (Range: 20-43 years) **Gender:** 4 males and 1 female  **Race/ Ethnicity:** NA  **Living arrangements:**  2 lived with their parents  1 lived independently  2 lived with others with learning difficulties  **Level of ID:** NA | Self-reports | Adults with DS valued family and friends, including those who helped them to join the mainstream education, work, housing, and friendship networks. Problems were attributed to negative attitudes and social barriers. |
| ^a^ Allahyari and Wolf-Branigin  (2018) [3]  United States of America | **Study Design:**  Phenomenological Qualitative research  **Data Collection:**  Structured face-to-face and telephone interviews | **Sampling technique:** NA  **Sample size:** n = 10  **Age:** Mean age of Adults with DS was NA (Range: 23+ years) **Gender:** Males and females; however, the numbers were NA  **Race/ Ethnicity:** Americans  **Living arrangements:** Some lived with family members. The numbers were NA.  **Level of ID:** NA | Proxy-reports (mothers and experts) | Most mothers were satisfied with the rehabilitation services. It was difficult to find a job and workers were paid less. Some of the jobs are shelter workshops where adults with DS are hidden at work, leading to exclusion from the community. |
| Brown  (1994) [4]  Canada | **Study Design:**  Quantitative longitudinal  **Data Collection:**  Survey | **Sampling technique:** NA  **Sample size:** n = 27  **Age:** Mean = 25 years for males (Range: 20-30) and 29 years for females (Range = 18-45 years)  **Gender:** 13 males and 14 females  **Race/ Ethnicity:** NA  **Living arrangements:** NA  **Level of ID:** NA | Self-reports (adults with DS) and proxy reports (sponsors) | Results revealed slow growth, decline, poor self-image, and concerns about range of leisure interests, with the intervention tending to stabilise or improve perceived achievement in adults with DS. |
| ^b^ Brown et al. (2001) [5]  Australia | **Study Design:**  Qualitative  **Data Collection:**  Interviews using vignettes | **Sampling technique:** NA  **Sample size:** n = 7  **Age:** Mean age = 51 years (Range: 50-52 years) **Gender:** 1 male and 1 female **Race/ Ethnicity:** NA  **Living arrangements:**  Lived with other people with disabilities but often within the community  **Level of ID:** NA | Self-reports (adults with DS) | Adults with DS had friends and were in relationships. They were happy, active and participated in some leisure activities. Privacy and family were crucial for the participants. They were independent but needed a certain level of support. |
| Bush and Tasse  (2017) [6]  United States of America | **Study Design:**  Quantitative cross-sectional  **Data Collection:**  Survey | **Sampling technique:** Random sampling  **Sample size:** n = 1857  **Age:** Mean age = 40.87 years (Range: 18-75 years) **Gender:** 1001 males and 856 females  **Race/ Ethnicity:**  0.5% American Indiana or Alaska Native  2.80% Asian  10.80% Black or African American  0.40% Pacific Islander  80.80% White  2.70% Other race not listed  1.30% Two or more races  0.60% Don’t know  **Living arrangements:** NA  **Level of ID:** Mild, moderate, severe and profound | Self-reports (adults with DS). Proxy reports were provided when adults with DS were unable to answer the survey questions. | 16% of adults with DS were employed in the community. Adults with DS worked fewer hours than other adults with IDD. In paid facility jobs, adults with DS received lower hourly wages than other adults with IDD. On average, adults with other IDD made the most decisions, followed by adults with DS, and then adults with ASD. |
| Cabeza-Ruiz et al. (2019) [7]  Spain | **Study Design:**  Quantitative cross-sectional  **Data Collection:**  Survey | **Sampling technique:** NA  **Sample size:** n = 38 were selected. (A female died during follow-up). So, the final sample size, n = 37  **Age:** Mean age = 37.57 years  (Range: 21- 58 years) **Gender:** 26 males and 11 females  **Race/ Ethnicity:** NA  **Living arrangements:**  All adults with DS lived in care centres for people with ID  **Level of ID:** Mild/moderate | Proxy reports (caregivers) | The intra-class correlation coefficient (ICC) was good and high (>0.80) for all tests, except for the 6-minute walk test, whose reliability was fair. Hence, SAMU-DISFIT Battery is shown to be a recommended physical fitness assessment tool in adults with DS to improve their QoL. |
| Camacho et al. (2021) [8]  Spain | **Study Design:**  Quantitative cross-sectional  **Data Collection:**  Survey | **Sampling technique:** Random sampling  **Sample size:** n = 39 (adults with DS)  **Age:** Mean age = 29 years (Range: 21-40 years) **Gender:** 24 males and 15 females  **Race/ Ethnicity:**  Spanish, Caucasian  **Living arrangements:**  All adults with DS lived with their families  **Level of ID:** Mild, moderate and severe | Self-reports (adults with DS) and proxy reports [informants (family members and teachers)] | Disagreements between the subgroups of adults with DS and informants indicated that results were higher in terms of perception for adults in the DS subgroup. Scores for all variables were higher in adults with DS who reported playing soccer competitively. |
| Carr (2008) [9]  United Kingdom | **Study Design:**  Quantitative longitudinal  **Data Collection:**  Survey | **Sampling technique:** NA  **Sample size:** Batch 1 (n = 38), Batch 2 (n = 37), Batch 3 (n= 34)  **Age:** Batch 1 (30 years), Batch 2 (35 years), Batch 3 (40 years) **Gender:** Batch 1 (20 males and 18 females), Batch 2 (20 males and 17 females), Batch 3 (17 males and 17 females)  **Race/ Ethnicity:** NA  **Living arrangements:**  Batch 1 (23 lived at home and 15 lived away from home)  Batch 2 (19 lived at home and 18 lived away from home)  Batch 3 (17 lived at home and 17 lived away from home)  **Level of ID:** NA in all the batches | Proxy reports (mothers and/ or caregivers) | Adults with DS were more restricted in their social relationships than their peers in the general population. Parents and caregivers restricted the independence of those they deemed less capable. |
| Collacott  (1992) [10]  United Kingdom | **Study Design:**  Quantitative cross-sectional  **Data Collection:**  Survey | **Sampling technique:** NA  **Sample size:** n = 308  **Age:** Mean age was NA (Range: over 18-60+ years) **Gender:** 166 males and 142 females  **Race/ Ethnicity:** NA  **Living arrangements:**  44 lived in hospital facilities  264 lived in community facilities  **Level of ID:** NA | Proxy reports (immediate carers) | Deterioration in most domains of ABS was statistically significant in adults with DS aged 50-59 and in all domains in those aged 60. Skill decline in older adults with DS is associated with ageing. |
| Dyke et al.  (2013) [11]  Western Australia | **Study Design:**  Qualitative  **Data Collection:**  Semi-structured interviews | **Sampling technique:** Purposive sampling  **Sample size:** n = 7  **Age:** Mean age = 21 years (Range: 19-27 years) **Gender:** 3 males and 4 females  **Race/ Ethnicity:** Australian  **Living arrangements:**  4 lived in family home  1 lived in family home/part-time supported accommodation  1 lived in supported accommodation  1 lived independently  **Level of ID:** NA | Proxy reports (mothers) | An unfulfilled potential of some young adults with DS was mothers decided to place their son or daughter in a supported workplace with other adults with a disability to ensure consistent work and safety. Most mothers described the QoL of the young adults with DS as “good.” |
| Faragher and Brown (2005)  [12]  Australia | **Study Design:**  Qualitative Case study  **Data Collection:**  Observations and interviews | **Sampling technique:** NA  **Sample size:** n = 5  **Age:** Mean age was NA (Range = 22-38 years) **Gender:** 1 male and 4 females  **Race/ Ethnicity:** NA  **Living arrangements:**  1 lived at home with family  1 lived independently  1 lived in a group home  For the remaining 2, this information is NA  **Level of ID:** NA | Self- reports (adults with DS) and proxy reports (carers or supervisors) | Numeracy skills are vital for adults with DS as they impact their QoL such as improving self-image. |
| Finkelstein et al. (2020) [13]  Israel | **Study Design:**  Qualitative  **Data Collection:**  Focus group and interviews | **Sampling technique:** NA  **Sample size:** n = 33  **Age:** Mean age for men = 39 years; Mean age for women = 35 years (Range = 30+ years) **Gender:** 14 males and 19 females  **Race/ Ethnicity:** NA  **Living arrangements:**  11 lived in their parents’ home (or in one case in the brother’s home)  22 lived in community residential settings or in designated facilities  **Level of ID:** NA | Self- reports (adults with DS) and proxy reports (family members or workplace supervisor) | It was difficult for adults with DS to talk about their own old age. In addition to practical and legal implications, the increased life expectancy of people with DS brings with it several personal and family challenges that adults with DS and their aging parents must contend with. |
| ^c^ Foley  (2013) [14]  United Kingdom | **Study Design:**  Qualitative  **Data Collection:**  Semi-structured interviews | **Sampling technique:** Purposive sampling  **Sample size:** n = 10  **Age:** Mean age of Adults with DS was NA (Range = 25+ years) **Gender:** NA for males and females  **Race/ Ethnicity:** NA  **Living arrangements:**  Adults with DS lived in parental home  **Level of ID:** NA | Proxy reports (parents) | Although parents agreed that their adults with DS had the same rights and needs as their peers with DS, many severely limit their adult son or daughter's life choices, particularly in regulating their son or daughter involved in a sexual relationship. All parents felt it was their duty to sometimes take the role of surrogate decision-maker for their adult with DS. |
| ^d^ Foley  (2014) [15]  United Kingdom | **Study Design:**  Qualitative  **Data Collection:**  Semi-structured interviews | **Sampling technique:** Purposive sampling  **Sample size:** n = 10  **Age:** Mean age of Adults with DS was NA (Range = 30+ years) **Gender:** NA for males and females  **Race/ Ethnicity:** NA  **Living arrangements:**  All adults with DS lived in parental home  **Level of ID:** NA | Proxy reports (mothers) | All mothers acknowledged that they subjected their adults with DS to a paternalistic regime of care, denying any causal link between this form of surveillance and the fact that, with one exception, none of their adults with DS had previously engaged in a sexual relationship. |
| Goldstein  (1988) [16]  Denmark | **Study Design:**  Quantitative cross-sectional  **Data Collection:** Interviews via survey | **Sampling technique:** NA  **Sample size:** n = 38  **Age:** Mean age = 45.7 years (Range: 40-50 years)  **Gender:** 23 males and 15 females  **Race/ Ethnicity:** Dutch  **Living arrangements:**  13% lived in private homes (parents/siblings)  82% lived in institutions  5% in sheltered residences with other people with IDD  **Level of ID:** NA | Proxy-reports (parent/sister or brother, staff members, personal social worker) | Adults with DS had high levels of recreation activities. Parents/ relatives were strongly advised to keep their adults with DS at home. However, the sheltered residence was successful if the best functioning among the adults with DS was chosen. |
| Heller et al. (2004) [17]  United States of America | **Study Design:**  Quantitative cross-sectional  **Data Collection:**  Survey | **Sampling technique:** NA  **Sample size:** n = 53  **Age:** Mean age = 39.72 years (Range: 30+ years)  **Gender:** 24 males and 29 females  **Race/ Ethnicity:**  32 Caucasian  17 African American  4 Hispanics  **Living arrangements:**  30 lived with family/ relatives  6 lived on their own  14 lived in small, supervised residence  3 lived in large, supervised residence  **Level of ID:** Mild and moderate | Self-reports (adults with DS) and proxy-reports (parent, other relative and staff) | Adults with DS in the training group showed significant improvement in attitudes toward exercise, exercise self-efficacy, expected outcomes, fewer cognitive–emotional barriers, life satisfaction, and marginally lower depression compared to the control group. |
| Jackson et al. (2014) [18]  United Kingdom | **Study Design:**  Qualitative  **Data Collection:**  Semi-structured interviews | **Sampling technique:** Purposive sampling  **Sample size:** n = 12  **Age:** Mean age was NA  (Range: 18-34 years)  **Gender:** 5 males and 7 females  **Race/ Ethnicity:** NA  **Living arrangements:**  8 lived with family  3 lived independently  2 lived with friends  1 lived in group home  The total count of living arrangements exceeded the number of Adults with DS as some had different living arrangements during the week and weekend. This led to more than 1 living arrangements reported.  **Level of ID:** NA | Self-reports (adults with DS) and proxy-reports (mothers) | Mood in adults with DS was found to link with communication. Communication difficulties led to frustration, anger, withdrawal or avoidance of situations, leading to loneliness. Frustration and low mood also affected communication. |
| Jevne et al. (2021) [19]  Norway | **Study Design:**  Qualitative  **Data Collection:**  Individual interviews | **Sampling technique:** Convenient sampling  **Sample size:** n = 8  **Age:** Mean age was 22 years  (Range: 22 years)  **Gender:** 3 males and 5 females  **Race/ Ethnicity:** Norwegian  **Living arrangements:**  5 lived with parents  1 lived partly with parents and partly in a sheltered residence  1 lived in an apartment with support  1 lived temporarily in a sheltered residence  **Level of ID:** NA | Self-reports (adults with DS) | QoL and subjective well-being of adults with DS were related to individualised support to improve independence at work, social leisure life and place of living. |
| Jobling et al.  (2000) [20]  Australia | **Study Design:**  Qualitative  **Data Collection:**  Discussion, whiteboard, videos | **Sampling technique:** NA  **Sample size:** n = 7  **Age:** Mean age was NA  (Range: 18-21 years)  **Gender:** 3 males and 4 females  **Race/ Ethnicity:** NA  **Living arrangements:**  All adults with DS lived at home with their families  **Level of ID:** Moderate | Self-reports (adults with DS) | Adults with DS enjoyed the six-session program to help them better understand friendships. In a group of learners, literacy needs were identified and there were issues in managing friendships and relationships that were part of the social dynamics of this group. |
| Kumin and Schoenbrodt  (2016) [21]  United States of America | **Study Design:**  Quantitative cross-sectional  **Data Collection:**  Online survey | **Sampling technique:** NA  **Sample size:** n = 95  **Age:** Mean age was NA  (Range: 18-61 years)  **Gender:** 53.2% males and 46.8% females  **Race/ Ethnicity:** NA  **Living arrangements:** NA  **Level of ID:** NA | Self-reports (adults with DS) and proxy reports (parents, siblings and support people) | High unemployment among adults with DS and current employment was limited to food, janitorial work, landscaping, and office work. A small percentage of adults with DS have full time paid employment. A combination of paid part-time work and unpaid volunteer work was common. |
| Landuran and N’Kaoua  (2021) [22]  France | **Study Design:**  Quantitative cross-sectional  **Data Collection:**  Mock-up: Semi-structured interview  Prototype 1 and 2: Semi-structured interview  Final version: Survey | **Sampling technique:** NA  **Sample size:** Mock-up (n = 12), Total for prototypes 1 and 2 (n = 20), Final version (n = 5)  **Age:** Mean age for Mock-up (30.2 years), Total for prototypes 1 and 2 (29.8 years), Final version (31 years)  Age range NA for all phases **Gender:** Mock-up (9 males and 3 females), Total for prototypes 1 and 2 (15 males and 5 females), Final version (2 males and 3 females)  **Race/ Ethnicity:** NA  **Living arrangements:**  Mock-up (8 lived with their parents and 4 lived independently)  Total for prototypes 1 and 2 (14 lived with their parents and 6 lived independently)  Final version – NA  **Level of ID:** NA | Self-reports (adults with DS) and proxy reports (parents and professionals) | Using digital assistant by adults with DS significantly improves the fullness of their life plan and well-being in the dimensions of autonomy. |
| Landuran et al. (2022) [23]  France | **Study Design:**  Quantitative cross-sectional  **Data Collection:**  Survey | **Sampling technique:** NA  **Sample size:** n = 18  **Age:** Mean age for group 1 = 33.3 years  (Range for group 1: 26-44 years)  Mean age for group 2 = 28 years  (Range for group 2: 21-41 years)  Mean age for group 3 = 28.8 years  (Range for group 3: 24-35 years)  **Gender:** 11 males and 7 females  **Race/ Ethnicity:** NA  **Living arrangements:**  11 lived with their families  7 lived independently  **Level of ID:** NA | Self-reports (adults with DS) | The most self-determined adults with DS have better skills in several aspects such as memory, motor and language skills, compared to those who are the least self-determined. |
| Landuran et al. (2022) [24]  France | **Study Design:**  Quantitative cross-sectional  **Data Collection:**  Survey | **Sampling technique:** NA  **Sample size:** n = 8  **Age:** Mean age was NA  (Range: 26-46 years)  **Gender:** 4 males and 4 females  **Race/ Ethnicity:** French  **Living arrangements:** All adults with DS lived alone in their home  **Level of ID:** NA | Self-reports (adults with DS) with supervision from proxies (caregivers) | The evaluation of a smart home for adults with DS showed impacts in most areas of their QoL such as promoting independence, living skills and self-esteem. |
| Li et al.  (2006) [25]  Hong Kong | **Study Design:**  Qualitative Ethnographic research  **Data Collection:**  Face-to-face interviews | **Sampling technique:** NA  **Sample size:** n = 8 (for only adults with DS aged ≥ 18 years)  **Age:** Mean age = 23.1 years  (Range: 18-31 years)  **Gender:** 3 males and 5 females  **Race/ Ethnicity:** Chinese  **Living arrangements:**  All adults with DS lived with their families  **Level of ID:** NA | Self-reports (adults with DS) and proxy-reports (parents, special schoolteachers, and staff members of non-governmental organisations) | Adults with DS demonstrated positive personality traits such as motivation to learn and try new things. Exposure to various extra-curricular activities and volunteer services also helped adults with DS to develop themselves. |
| Love and Agiovlasitis  (2016) [26]  United States of America | **Study Design:** Qualitative Grounded-theory approach  **Data Collection:**  Interviews | **Sampling technique:** Purposive sampling  **Sample size:** n = 30  **Age:** Mean age = 43.87 years  (Range: 18-71 years)  **Gender:** 12 males and 18 females  **Race/ Ethnicity:** NA  **Living arrangements:**  5 lived at home with family members  13 lived in small-scale group homes  12 lived in larger institutions  **Level of ID:** NA | Self-reports (adults with DS) | In this study, adults with DS expressed a general enjoyment of a wide range of exercise, sports, and physical activities and should be considered when designing programs to improve their QoL. |
| Mihaila et al.  (2020) [27]  United States of America | **Study Design:**  Quantitative cross-sectional  **Data Collection:**  Survey | **Sampling technique:** NA  **Sample size:** n = 44  **Age:** Mean age = 37.56 years  (Range: 25–56 years)  **Gender:** 23 males and 21 females  **Race/ Ethnicity:**  44 White non-Hispanic  **Living arrangements:**  30 lived in family home  3 lived in group home  3 lived in supported apartment  8 lived independently  **Level of ID:** NA | Self-reports (adults with DS) and proxy-reports (caregivers) | Most adults with DS did not meet required recommendations for leisure-time physical activity intensity (i.e., 150 minutes/ week moderately active activity) and did not exceed levels of leisure such as watching television, found in the general population (i.e., 2–3 hour/day). Adults with DS are self-initiated and self-engaged in most of their leisure activities. |
| Mihaila et al.  (2017) [28]  United States of America | **Study Design:**  Quantitative cross-sectional  **Data Collection:**  Survey | **Sampling technique:** NA  **Sample size:** n = 62  **Age:** Mean age = 37.82 years  (Range: 30–53 years)  **Gender:** 36 males and 26 females  **Race/ Ethnicity:**  62 White non-Hispanic  **Living arrangements:**  42 lived in family home  7 lived in group home  13 lived independently with support  **Level of ID:** NA | Proxy-reports (primary caregiver which were mainly parents) | Middle-aged and older adults with DS frequently engaged in social and passive leisure activities, with low participation in physically and mentally stimulating leisure activities. |
| Pérez et al.  (2018) [29]  Spain | **Study Design:**  Quantitative cross-sectional (Pilot study)  **Data Collection:**  Survey | **Sampling technique:** NA  **Sample size:** n = 14  **Age:** Mean age = 37.7 years (Range: 21-49 years)  **Gender:** 7 males and 7 females  **Race/ Ethnicity:** Spanish  **Living arrangements:** NA  **Level of ID:** Moderate and severe | Self-reports [adults with DS (with the help and under the supervision of parents/ caregivers)] | There was no statistical significance in the improvement in swimming technique performed twice weekly for 12 weeks on the QoL of adults with DS (p>0.05). |
| Robles-Bello et al. (2022) [30]  Spain | **Study Design:**  Quantitative two-stage cross-sectional study  **Data Collection:**  Survey | **Sampling technique:** Random sampling  **Sample size:** n = 196  **Age:** Mean age = 22 years (Range: 20-25 years)  **Gender:** 7 males and 7 females  **Race/ Ethnicity:** Spanish  **Living arrangements:** NA  **Level of ID:** NA | Self-reports (adults with DS) | A new version of EQ-i:YV was a valid and reliable tool to measure emotional intelligence in Spanish adults with DS in 5 factors: general mood, stress management, adaptability, interpersonal and intrapersonal. |
| **^e^** Roll and Bowers  (2019) [31]  United States of America | **Study Design:**  Qualitative  **Data Collection:**  Interviews | **Sampling technique:** Theoretical sampling  **Sample size:** n = 29  **Age:** Mean age of Adults with DS = 31.2 years (Range: 19-58 years)  **Gender:** NA for males and females  **Race/ Ethnicity:**  Mostly White  **Living arrangements:**  Some adults with DS lived in family home while other Adults with DS independently.  **Level of ID:** Mild or moderate | Proxy reports (family members) | Using building and connecting approaches, family members met the social needs of adults with DS. The conditions that influenced whether family members used either approach included family values and attitudes, trust, resources and opportunities. |
| Roll and Koehly  (2020) [32]  United States of America | **Study Design:**  Mixed methods  **Data Collection:**  Quantitative and qualitative data | **Sampling technique:** NA  **Sample size:** n = 27  **Age:** Mean age = 31.2 years (Range: 19-58 years)  **Gender:** 8 males and 19 females  **Race/ Ethnicity:**  White  **Living arrangements:**  18 lived with family members  9 lived without family members  **Level of ID:** Mild or moderate | Self-reports (adults with DS) and proxy reports (family members) | The self-reported total network size of adults with DS was significantly smaller than the network size based on proxy reports. There were significant differences between self- and proxy-reported networks for most relationship groups. Work was shown to be an important way for adults with DS to build and maintain close relationships. |
| Sánchez-Teruel et al. (2020) [33]  Spain | **Study Design:**  Quantitative cross-sectional  **Data Collection:**  Survey | **Sampling technique:** NA  **Sample size:** n = 742  **Age:** Mean age = 26.04 years (Range: 24-32 years)  **Gender:** 409 males and 333 females  **Race/ Ethnicity:**  Spanish  **Living arrangements:** NA  **Level of ID:** Mild | Self-reports (adults with DS). Some Adults with DS who displayed difficulties were supported by the collaborating psychologists, but this case was rare. | A new scale named EQ-i: SVDS for adults with DS was obtained with a structure of 4 factors: mood, stress management, interpersonal, and intrapersonal. This new scale was reduced to 25 items. Goodness-of-fit indices were excellent (RMSEA [95% CI] = 02[0.01; 0.03]; CFI = 0.99; TLI = 0.98; GFI = 0.87; AGFI = 0.89). The internal consistency of the four factors and the calculated total score resulted in high values. |
| Schroeder-Kurth et al.  (1990) [34]  Germany | **Study Design:**  Quantitative cross-sectional  **Data Collection:**  Survey | **Sampling technique:** NA  **Sample size:** n = 118  **Age:** Mean age was NA  (Range: 18 -53years)  **Gender:** 65 males and 53 females  **Race/ Ethnicity:** NA  **Living arrangements:**  59 lived under family care  59 lived under institutional care  **Level of ID:** NA | Proxy reports (parents and educators) | There was a significant difference between family-care and institutionalised adults with DS. Results showed that adults with DS in family-care were more oriented, skilled and productive, emotionally stable and less disruptive in their behaviour than those in institutionalised care (p≤0.05). |
| Scott et al.  (2014) [35]  Australia | **Study Design:**  Qualitative  **Data Collection:**  Individual and focus group discussions | **Sampling technique:** Purposive and convenience sampling  **Sample size:** n = 12  **Age:** Mean age = 21 years  (Range: 18-29 years)  **Gender:** 6 males and 6 females  **Race/ Ethnicity:** NA  **Living arrangements:**  10 lived at home with parents  1 lived with a friend  1 lived independently near to parents’ house  **Level of ID:** NA | Self-reports (adults with DS) | Relationships played a crucial role in ensuring a good life for young adults with DS. Participants were actively involved in their communities and desired independent living. |
| Thompson et al. (2020) [36]  United States of America | **Study Design:**  Qualitative  **Data Collection:**  Photo-elicitation, open-ended case study interview, focused case study interview, direct observations | **Sampling technique:** Purposive sampling through convenience and snowball sampling methods  **Sample size:** n = 4  **Age:** Mean age was NA  (Range: 21-39 years)  **Gender:** 2 males and 2 females  **Race/ Ethnicity:**  1 Caucasian  1 Biracial- African American and Caucasian  1 Caucasian  1 Hispanic/Latino  **Living arrangements:**  2 lived with parents  2 lived with independently  **Level of ID:** NA | Self-reports (adults with DS) and proxy reports (parents, siblings, college professor and work supervisor) | Adults with DS demonstrated rich, complex, and meaningful lives filled with joyful and reciprocal relationships and increasing independence throughout adulthood. From a thriving perspective, a DS diagnosis does not prevent the individual from enjoying life or improving the lives of those around them. |
| Thomson et al. (1995) [37]  United Kingdom | **Study Design:**  Mixed methods  **Data Collection:**  Longitudinal with case studies | **Sampling technique:** NA  **Sample size:** n = 35 (One died between the 2^nd^ and 3^rd^ transition points of the study (finally, n = 34 adults with DS). Although, 17 and 2 Adults with DS were reported in the quantitative and qualitative strands, respectively  **Age:** Mean = 22 years (Range: NA)  **Gender:** 10 males and 24 females  **Race/ Ethnicity:** NA  **Living arrangements:**  30 lived with parents  2 lived in residential care  For the remaining 2, this information is NA  **Level of ID:** Mild/moderate, severe and profound | Self-reports (adults with DS) and proxy reports (parents and staff) | A significant proportion of adults with DS depended primarily on their parents as caregivers. The outcome of the transition from school to adulthood was disappointing as all have followed a similar path from a special school to a variety of adult training or resource centres. |
| van Heumen and Schippers (2016) [38]  The Netherlands | **Study Design:**  Qualitative - Fourth generation evaluation method principles  **Data Collection:**  Semi-structured interviews, focus groups, photovoice | **Sampling technique:** NA  **Sample size:** n = 4  **Age:** Mean = 27 years (Range: 26-28 years)  **Gender:** 4 males and 0 female  **Race/ Ethnicity:** Dutch  **Living arrangements:**  All Adults with DS lived in their own apartments with support from residential setting  **Level of ID:** Not severe | Self-reports (adults with DS) and proxy reports (family caregivers) | Families appreciated participation in the future planning project. Caregivers spoke about the project’s impact on the QoL of the young adults with DS in becoming more independent and determined throughout the transition process. However, the young adults with DS had several threats to their independence and inclusion in their homes and community. |
| Villani et al. (2020) [39]  Italy | **Study Design:**  Quantitative cross-sectional  **Data Collection:**  Survey | **Sampling technique:** NA  **Sample size:** n = 46  **Age:** Mean = 40.6 years (Range: ≥ 18 years)  **Gender:** 23 males and 23 females  **Race/ Ethnicity:** NA  **Living arrangements:**  30 lived with parents or guardians  7 lived with siblings  9 lived with non-relatives  **Level of ID:** Not severe | Self-reports (adults with DS) and proxy reports (parents or siblings or caregivers) | In the pre-lockdown period of COVID-19, adults with DS had a significant deterioration over time (i.e., per year) for the Depression Rating Scale score (β = 0.55; 95% CI 0.34; 0.76). In the post-lockdown period, a significant deterioration in social withdrawal (β = 3.05, 95% CI 0.39; 5.70), instrumental activities of daily living (β = 1.13, 95% CI 0.08; 2.18) and depression rating (β = 1.65, 95% CI 0.33; 2.97) scales scores were observed, and a significant improvement in aggressive behaviour (β = −1.40, 95% CI −2.69; −0.10). |

**Note:** ^a^ = the study only presented the sample size of mothers and experts; ^b^ = the study presented vignettes for only 2 participants out of the 7 adults with Down syndrome;

^c^ = the study only presented the sample size of parents, and not adults with Down syndrome; ^d^ = the study only presented the sample size of mothers, and not adults with Down syndrome; ^e^ = the study only presented the sample size of family members, and not adults with Down syndrome; ABS = Adaptive Behaviour Scale; AGFI =Adjusted

goodness-of-fit index; ASD = Autism spectrum disorder; COVID-19 = Coronavirus disease 19; CDI-S = Children’s Depression Inventory Short Form; CFI = Comparative

fit index; CI = Confidence interval; DS = Down syndrome; EQ-i: SVDS = Emotional Quotient Inventory: Short Version for Down Syndrome; EQ-i:YV = Bar-On Emotional Quotient Inventory: Youth Version; GFI = Goodness-of-fit index; ID = Intellectual disability; IDD = Intellectual and developmental disability; n = sample size; NA = Not available; PIMRA-AD = Psychopathology Instrument for Mentally Retarded Adults Affective Disorders Subscale; QoL= Quality of Life; RMSEA = Root mean square error of approximation; SAMU-DISFIT = Servicios de Asistencia Médica de Urgencias Disability Fitness Battery; TLI = Tucker Lewis Index.

**References**

1. Ailey SH, Miller AM, Heller T, Smith Jr EV. Evaluating an interpersonal model of depression among adults with Down syndrome. Research and Theory for Nursing Practice. 2006;20(3):229-46.

2. Alderson P. Down's syndrome: cost, quality and value of life. Social science & medicine. 2001;53(5):627-38.

3. Allahyari T, Wolf-Branigin M. Quality of life of adults with Down syndrome in Virginia. Journal of Down Syndrome & Chromosome Abnormalities. 2018;4(1):2472-1115.

4. Brown R. Down Syndrome and quality of life: some challenges for future practice. Down Syndrome Research and Practice. 1994;2(1):19-30.

5. Brown R, Taylor J, Matthews B. Quality of life-ageing and Down syndrome. Down Syndrome Research and Practice. 2001;6(3):111-6.

6. Bush KL, Tassé MJ. Employment and choice-making for adults with intellectual disability, autism, and Down syndrome. Research in Developmental Disabilities. 2017;65:23-34.

7. Cabeza-Ruiz R, Alcántara-Cordero FJ, Ruiz-Gavilán I, Sánchez-López AM. Feasibility and reliability of a physical fitness test battery in individuals with Down syndrome. International Journal of Environmental Research and Public Health. 2019;16(15):2685.

8. Camacho R, Castejón-Riber C, Requena F, Camacho J, Escribano BM, Gallego A, et al. Quality of life: changes in self-perception in people with Down syndrome as a result of being part of a football/soccer team. Self-reports and external reports. Brain Sciences. 2021;11(2):226.

9. Carr J. The everyday life of adults with Down syndrome. Journal of Applied Research in Intellectual Disabilities. 2008;21(5):389-97.

10. Collacott RA. The effect of age and residential placement on adaptive behaviour of adults with Down's syndrome. The British Journal of Psychiatry. 1992;161(5):675-9.

11. Dyke P, Bourke J, Llewellyn G, Leonard H. The experiences of mothers of young adults with an intellectual disability transitioning from secondary school to adult life. Journal of Intellectual and developmental Disability. 2013;38(2):149-62.

12. Faragher R, Brown R. Numeracy for adults with Down syndrome: it's a matter of quality of life. Journal of Intellectual Disability Research. 2005;49(10):761-5.

13. Finkelstein A, Tenenbaum A, Bachner YG. ‘I will never be old’: adults with Down syndrome and their parents talk about ageing-related challenges. Ageing & Society. 2020;40(8):1788-807.

14. Foley S. Reluctant ‘Jailors’ speak out: parents of adults with Down syndrome living in the parental home on how they negotiate the tension between empowering and protecting their intellectually disabled sons and daughters. British Journal of Learning Disabilities. 2013;41(4):304-11.

15. Foley S. A Foucauldian Reading of Mothers' Views on the Paternalism/Autonomy Debate in Relation to the Sexual Practices of Their Intellectually Disabled Adult Sons and Daughters. Irish Journal of Sociology. 2014;22(2):64-85.

16. Goldstein H. Living conditions of an adult population with Down's syndrome. Research in developmental disabilities. 1988;9(2):123-34.

17. Heller T, Hsieh K, Rimmer JH. Attitudinal and psychosocial outcomes of a fitness and health education program on adults with Down syndrome. American Journal on Mental Retardation. 2004;109(2):175-85.

18. Jackson C, Cavenagh P, Clibbens J. Communication and self‐esteem in adults with Down syndrome. International journal of language & communication disorders. 2014;49(3):275-87.

19. Jevne KW, Kollstad M, Dolva A-S. The perspective of emerging adults with Down syndrome–On quality of life and well-being. Journal of Intellectual Disabilities. 2021:17446295211030097.

20. Jobling A, Moni KB, Nolan A. Understanding friendship: Young adults with Down syndrome exploring relationships. Journal of Intellectual and Developmental Disability. 2000;25(3):235-45.

21. Kumin L, Schoenbrodt L. Employment in adults with Down syndrome in the United States: results from a national survey. Journal of Applied Research in Intellectual Disabilities. 2016;29(4):330-45.

22. Landuran A, N’Kaoua B. Designing a digital assistant for developing a life plan. International Journal of Human–Computer Interaction. 2021;37(18):1749-59.

23. Landuran A, Raynaud C, N’kaoua B. Cognitive and Motor Skills of People with Down Syndrome According to Their Perceived Self-Determination. Journal of Developmental and Physical Disabilities. 2022;34(1):89-111.

24. Landuran A, Sauzéon H, Consel C, N’Kaoua B. Evaluation of a smart home platform for adults with Down syndrome. Assistive Technology. 2022:1-11. doi: 10.1080/10400435.2022.2075487.

25. Li EP-Y, Liu Y-m, Lok NC-y, Lee VW-k. Successful experience of people with Down syndrome. Journal of Intellectual Disabilities. 2006;10(2):143-54.

26. Love A, Agiovlasitis S. How do adults with Down syndrome perceive physical activity? Adapted Physical Activity Quarterly. 2016;33(3):253-70.

27. Mihaila I, Handen BL, Christian BT, Hartley SL. Leisure activity in middle‐aged adults with Down syndrome: Initiators, social partners, settings and barriers. Journal of Applied Research in Intellectual Disabilities. 2020;33(5):865-75.

28. Mihaila I, Hartley SL, Handen BL, Bulova PD, Tumuluru RV, Devenny DA, et al. Leisure activity and caregiver involvement in middle-aged and older adults with Down syndrome. Intellectual and developmental disabilities. 2017;55(2):97-109.

29. Pérez CA, Carral JMC, Costas AÁ, Martínez SV, Martínez-Lemos RI. Water-based exercise for adults with Down syndrome: Findings from a preliminary study. International Journal of Therapy and Rehabilitation. 2018;25(1):20-8.

30. Robles-Bello MA, Sánchez-Teruel D, Valencia Naranjo N, Delgado Rodríguez R. Preliminary Study on Emotional Competence in Adults with Down Syndrome. International Journal of Disability, Development and Education. 2020:1-19.

31. Roll AE, Bowers BJ. Building and connecting: family strategies for developing social support networks for adults with Down syndrome. Journal of family nursing. 2019;25(1):128-51.

32. Roll AE, Koehly LM. One social network, two perspectives: Social networks of people with Down syndrome based on self‐reports and proxy reports. Journal of Applied Research in Intellectual Disabilities. 2020;33(6):1188-98.

33. Sánchez-Teruel D, Robles-Bello MA, Camacho-Conde JA. Assessment of emotional intelligence in adults with Down syndrome: Psychometric properties of the Emotional Quotient Inventory. PLoS ONE. 2020;15(7):e0236087.

34. Schroeder‐Kurth T, Schaffert G, Koeckritz W, Kernich M. Quality of life of adults with trisomy 21 living in mental retardation homes compared with those staying under parental care. American Journal of Medical Genetics. 1990;37(S7):317-21.

35. Scott M, Foley K-R, Bourke J, Leonard H, Girdler S. “I have a good life”: the meaning of well-being from the perspective of young adults with Down syndrome. Disability and Rehabilitation. 2014;36(15):1290-8.

36. Thompson T, Talapatra D, Hazel CE, Coleman J, Cutforth N. Thriving with Down syndrome: A qualitative multiple case study. Journal of Applied Research in Intellectual Disabilities. 2020;33(6):1390-404.

37. Thomson GO, Ward KM, Wishart JG. The transition to adulthood for children with Down's syndrome. Disability & Society. 1995;10(3):325-40.

38. van Heumen L, Schippers A. Quality of life for young adults with intellectual disability following individualised support: Individual and family responses. Journal of Intellectual & Developmental Disability. 2016;41(4):299-310.

39. Villani ER, Vetrano DL, Damiano C, Paola AD, Ulgiati AM, Martin L, et al. Impact of COVID-19-related lockdown on psychosocial, cognitive, and functional well-being in adults with down syndrome. Frontiers in Psychiatry. 2020;11:578686.
