# Supplementary material for: Quality of life in adults with Down syndrome: A mixed methods systematic review
Source: PLoS One. 2023 May 1;18(5):e0280014. doi: 10.1371/journal.pone.0280014 (PMC10150991; doi:10.1371/journal.pone.0280014)
Supplement: S3 Table — (DOCX) [file pone.0280014.s004.docx]

**S3 Table.** **Quality assessment of included studies (quality appraisal)**

| **Reference** | **1** | **2** | **3** | **4** | **5** | **6** | **7** | **8** | **9** | **10** | **11** | **12** | **13** | **Total  score** | **Percentage (%)** |
| --- | --- | --- | --- | --- | --- | --- | --- | --- | --- | --- | --- | --- | --- | --- | --- |
| Ailey et al. (2006) [1] | 3 | 3 | 1 | 3 | 0 | 3 | 2 | 3 | 1 | 2 | 3 | 0 | 2 | 26 | 66.67 |
| Alderson (2001) [2] | 2 | 3 | 2 | 3 | 0 | 2 | 3 | 2 | 2 | 2 | 2 | 0 | 1 | 24 | 61.54 |
| Allahyari and Wolf-Branigin (2018) [3] | 3 | 3 | 3 | 3 | 0 | 2 | 3 | 2 | 1 | 2 | 3 | 2 | 2 | 29 | 74.36 |
| Brown (1994) [4] | 0 | 3 | 2 | 3 | 0 | 2 | 2 | 2 | 0 | 2 | 2 | 1 | 1 | 20 | 51.28 |
| Brown et al. (2001) [5] | 3 | 3 | 1 | 3 | 0 | 2 | 3 | 2 | 1 | 2 | 2 | 2 | 0 | 24 | 61.54 |
| Bush and Tasse (2017) [6] | 1 | 3 | 3 | 3 | 2 | 0 | 2 | 2 | 2 | 1 | 2 | 0 | 2 | 23 | 58.97 |
| Cabeza-Ruiz et al. (2019) [7] | 0 | 3 | 3 | 3 | 3 | 2 | 3 | 2 | 3 | 1 | 3 | 0 | 3 | 29 | 74.36 |
| Camacho et al. (2021) [8] | 3 | 3 | 3 | 3 | 0 | 3 | 3 | 3 | 1 | 2 | 2 | 0 | 2 | 28 | 71.79 |
| Carr (2008) [9] | 0 | 3 | 2 | 2 | 1 | 2 | 2 | 2 | 2 | 1 | 2 | 0 | 1 | 20 | 51.28 |
| Collacott (1992) [10] | 0 | 3 | 3 | 2 | 1 | 2 | 1 | 2 | 2 | 2 | 2 | 0 | 1 | 21 | 53.85 |
| Dyke et al. (2013) [11] | 3 | 3 | 3 | 3 | 3 | 2 | 3 | 3 | 3 | 2 | 3 | 0 | 2 | 33 | 84.62 |
| Faragher and Brown (2005) [12] | 2 | 3 | 3 | 3 | 0 | 2 | 2 | 2 | 1 | 1 | 2 | 0 | 1 | 22 | 56.41 |
| Finkelstein et al. (2020) [13] | 0 | 3 | 3 | 3 | 1 | 3 | 3 | 3 | 1 | 3 | 3 | 1 | 3 | 30 | 76.92 |
| Foley (2013) [14] | 1 | 3 | 3 | 3 | 2 | 3 | 3 | 2 | 1 | 3 | 1 | 0 | 2 | 27 | 69.23 |
| Foley (2014) [15] | 3 | 3 | 3 | 3 | 2 | 3 | 3 | 2 | 1 | 3 | 3 | 0 | 2 | 31 | 79.49 |
| Goldstein (1988) [16] | 1 | 3 | 3 | 2 | 1 | 1 | 2 | 2 | 2 | 0 | 0 | 0 | 2 | 19 | 48.72 |
| Heller et al. (2004) [17] | 3 | 3 | 3 | 3 | 0 | 3 | 2 | 3 | 3 | 3 | 3 | 0 | 2 | 31 | 79.49 |
| Jackson et al. (2014) [18] | 3 | 3 | 3 | 3 | 3 | 2 | 2 | 2 | 2 | 1 | 3 | 0 | 1 | 28 | 71.79 |
| Jevne et al. (2021) [19] | 2 | 3 | 3 | 2 | 3 | 3 | 3 | 3 | 3 | 1 | 2 | 0 | 2 | 30 | 76.92 |
| Jobling et al. (2000) [20] | 1 | 3 | 3 | 1 | 0 | 2 | 2 | 2 | 1 | 2 | 1 | 0 | 1 | 19 | 48.72 |
| Kumin and Schoenbrodt (2016) [21] | 2 | 3 | 3 | 3 | 1 | 3 | 3 | 3 | 2 | 1 | 2 | 0 | 1 | 27 | 69.23 |
| Landuran and N’Kaoua (2021) [22] | 3 | 3 | 3 | 3 | 0 | 3 | 3 | 3 | 2 | 3 | 3 | 2 | 2 | 33 | 84.62 |
| Landuran et al. (2022) [23] | 3 | 3 | 3 | 3 | 0 | 3 | 3 | 3 | 2 | 3 | 3 | 2 | 2 | 33 | 84.62 |
| Landuran et al. (2022) [24] | 3 | 3 | 3 | 3 | 0 | 3 | 3 | 3 | 1 | 3 | 2 | 2 | 2 | 31 | 79.49 |
| Li et al. (2006) [25] | 1 | 3 | 3 | 3 | 0 | 3 | 2 | 3 | 1 | 0 | 3 | 0 | 0 | 22 | 56.41 |
| Love and Agiovlasitis (2016) [26] | 1 | 3 | 3 | 3 | 3 | 2 | 2 | 3 | 2 | 3 | 3 | 0 | 2 | 30 | 76.92 |
| Mihaila et al. (2020) [27] | 2 | 3 | 3 | 2 | 0 | 2 | 3 | 3 | 2 | 2 | 3 | 0 | 2 | 27 | 69.23 |
| Mihaila et al. (2017) [28] | 1 | 3 | 3 | 1 | 0 | 2 | 3 | 3 | 2 | 2 | 3 | 0 | 2 | 25 | 64.10 |
| Pérez et al. (2018) [29] | 1 | 3 | 3 | 3 | 3 | 3 | 2 | 2 | 1 | 1 | 2 | 3 | 2 | 29 | 74.36 |
| Robles-Bello et al. (2022) [30] | 3 | 3 | 3 | 3 | 2 | 3 | 3 | 3 | 1 | 3 | 3 | 3 | 2 | 35 | 89.74 |
| Roll and Bowers (2019) [31] | 1 | 3 | 2 | 3 | 3 | 3 | 3 | 3 | 1 | 3 | 3 | 0 | 3 | 31 | 79.49 |
| Roll and Koehly (2020) [32] | 2 | 3 | 2 | 3 | 0 | 3 | 3 | 3 | 1 | 3 | 3 | 0 | 2 | 28 | 71.79 |
| Sánchez-Teruel et al. (2020) [33] | 2 | 3 | 3 | 3 | 0 | 3 | 3 | 3 | 3 | 2 | 3 | 0 | 3 | 31 | 79.49 |
| Schroeder-Kurth et al. (1990) [34] | 0 | 3 | 2 | 3 | 0 | 1 | 3 | 1 | 1 | 2 | 2 | 0 | 0 | 18 | 46.15 |
| Scott et al. (2014) [35] | 2 | 3 | 3 | 3 | 3 | 3 | 3 | 3 | 2 | 3 | 3 | 0 | 2 | 33 | 84.62 |
| Thompson et al. (2020) [36] | 3 | 3 | 2 | 3 | 3 | 3 | 3 | 3 | 1 | 3 | 3 | 0 | 2 | 32 | 82.05 |
| Thomson et al. (1995) [37] | 1 | 3 | 3 | 2 | 0 | 1 | 2 | 1 | 2 | 0 | 1 | 0 | 0 | 16 | 41.03 |
| van Heumen and Schippers (2016) [38] | 3 | 3 | 3 | 3 | 0 | 3 | 3 | 3 | 2 | 2 | 3 | 0 | 2 | 30 | 76.92 |
| Villani et al. (2020) [39] | 1 | 3 | 3 | 2 | 0 | 3 | 2 | 2 | 1 | 1 | 2 | 0 | 2 | 22 | 56.41 |

**Note:** 1. Theoretical or conceptual underpinning to the research. 2. Statement of research aim(s). 3. Clear description of research setting and target population. 4. The study design is appropriate to address the stated research aim(s). 5. Appropriate sampling to address the research aim(s). 6. Rationale for choice of data collection tool(s). 7. The format and content of data collection tool are appropriate to address the stated research aim(s). 8. Description of data collection procedure. 9. Recruitment data provided. 10. Justification of analytic method selected. 11. The method of analysis was appropriate to answer the research aim(s). 12. Evidence that the research stakeholders have been considered in research design or conduct. 13. Strengths and limitations critically discussed.

**References**

1. Ailey SH, Miller AM, Heller T, Smith Jr EV. Evaluating an interpersonal model of depression among adults with Down syndrome. Research and Theory for Nursing Practice. 2006;20(3):229-46.

2. Alderson P. Down's syndrome: cost, quality and value of life. Social science & medicine. 2001;53(5):627-38.

3. Allahyari T, Wolf-Branigin M. Quality of life of adults with Down syndrome in Virginia. Journal of Down Syndrome & Chromosome Abnormalities. 2018;4(1):2472-1115.

4. Brown R. Down Syndrome and quality of life: some challenges for future practice. Down Syndrome Research and Practice. 1994;2(1):19-30.

5. Brown R, Taylor J, Matthews B. Quality of life-ageing and Down syndrome. Down Syndrome Research and Practice. 2001;6(3):111-6.

6. Bush KL, Tassé MJ. Employment and choice-making for adults with intellectual disability, autism, and Down syndrome. Research in Developmental Disabilities. 2017;65:23-34.

7. Cabeza-Ruiz R, Alcántara-Cordero FJ, Ruiz-Gavilán I, Sánchez-López AM. Feasibility and reliability of a physical fitness test battery in individuals with Down syndrome. International Journal of Environmental Research and Public Health. 2019;16(15):2685.

8. Camacho R, Castejón-Riber C, Requena F, Camacho J, Escribano BM, Gallego A, et al. Quality of life: changes in self-perception in people with Down syndrome as a result of being part of a football/soccer team. Self-reports and external reports. Brain Sciences. 2021;11(2):226.

9. Carr J. The everyday life of adults with Down syndrome. Journal of Applied Research in Intellectual Disabilities. 2008;21(5):389-97.

10. Collacott RA. The effect of age and residential placement on adaptive behaviour of adults with Down's syndrome. The British Journal of Psychiatry. 1992;161(5):675-9.

11. Dyke P, Bourke J, Llewellyn G, Leonard H. The experiences of mothers of young adults with an intellectual disability transitioning from secondary school to adult life. Journal of Intellectual and developmental Disability. 2013;38(2):149-62.

12. Faragher R, Brown R. Numeracy for adults with Down syndrome: it's a matter of quality of life. Journal of Intellectual Disability Research. 2005;49(10):761-5.

13. Finkelstein A, Tenenbaum A, Bachner YG. ‘I will never be old’: adults with Down syndrome and their parents talk about ageing-related challenges. Ageing & Society. 2020;40(8):1788-807.

14. Foley S. Reluctant ‘Jailors’ speak out: parents of adults with Down syndrome living in the parental home on how they negotiate the tension between empowering and protecting their intellectually disabled sons and daughters. British Journal of Learning Disabilities. 2013;41(4):304-11.

15. Foley S. A Foucauldian Reading of Mothers' Views on the Paternalism/Autonomy Debate in Relation to the Sexual Practices of Their Intellectually Disabled Adult Sons and Daughters. Irish Journal of Sociology. 2014;22(2):64-85.

16. Goldstein H. Living conditions of an adult population with Down's syndrome. Research in developmental disabilities. 1988;9(2):123-34.

17. Heller T, Hsieh K, Rimmer JH. Attitudinal and psychosocial outcomes of a fitness and health education program on adults with Down syndrome. American Journal on Mental Retardation. 2004;109(2):175-85.

18. Jackson C, Cavenagh P, Clibbens J. Communication and self‐esteem in adults with Down syndrome. International journal of language & communication disorders. 2014;49(3):275-87.

19. Jevne KW, Kollstad M, Dolva A-S. The perspective of emerging adults with Down syndrome–On quality of life and well-being. Journal of Intellectual Disabilities. 2021:17446295211030097.

20. Jobling A, Moni KB, Nolan A. Understanding friendship: Young adults with Down syndrome exploring relationships. Journal of Intellectual and Developmental Disability. 2000;25(3):235-45.

21. Kumin L, Schoenbrodt L. Employment in adults with Down syndrome in the United States: results from a national survey. Journal of Applied Research in Intellectual Disabilities. 2016;29(4):330-45.

22. Landuran A, N’Kaoua B. Designing a digital assistant for developing a life plan. International Journal of Human–Computer Interaction. 2021;37(18):1749-59.

23. Landuran A, Raynaud C, N’kaoua B. Cognitive and Motor Skills of People with Down Syndrome According to Their Perceived Self-Determination. Journal of Developmental and Physical Disabilities. 2022;34(1):89-111.

24. Landuran A, Sauzéon H, Consel C, N’Kaoua B. Evaluation of a smart home platform for adults with Down syndrome. Assistive Technology. 2022:1-11. doi: 10.1080/10400435.2022.2075487.

25. Li EP-Y, Liu Y-m, Lok NC-y, Lee VW-k. Successful experience of people with Down syndrome. Journal of Intellectual Disabilities. 2006;10(2):143-54.

26. Love A, Agiovlasitis S. How do adults with Down syndrome perceive physical activity? Adapted Physical Activity Quarterly. 2016;33(3):253-70.

27. Mihaila I, Handen BL, Christian BT, Hartley SL. Leisure activity in middle‐aged adults with Down syndrome: Initiators, social partners, settings and barriers. Journal of Applied Research in Intellectual Disabilities. 2020;33(5):865-75.

28. Mihaila I, Hartley SL, Handen BL, Bulova PD, Tumuluru RV, Devenny DA, et al. Leisure activity and caregiver involvement in middle-aged and older adults with Down syndrome. Intellectual and developmental disabilities. 2017;55(2):97-109.

29. Pérez CA, Carral JMC, Costas AÁ, Martínez SV, Martínez-Lemos RI. Water-based exercise for adults with Down syndrome: Findings from a preliminary study. International Journal of Therapy and Rehabilitation. 2018;25(1):20-8.

30. Robles-Bello MA, Sánchez-Teruel D, Valencia Naranjo N, Delgado Rodríguez R. Preliminary Study on Emotional Competence in Adults with Down Syndrome. International Journal of Disability, Development and Education. 2020:1-19.

31. Roll AE, Bowers BJ. Building and connecting: family strategies for developing social support networks for adults with Down syndrome. Journal of family nursing. 2019;25(1):128-51.

32. Roll AE, Koehly LM. One social network, two perspectives: Social networks of people with Down syndrome based on self‐reports and proxy reports. Journal of Applied Research in Intellectual Disabilities. 2020;33(6):1188-98.

33. Sánchez-Teruel D, Robles-Bello MA, Camacho-Conde JA. Assessment of emotional intelligence in adults with Down syndrome: Psychometric properties of the Emotional Quotient Inventory. PLoS ONE. 2020;15(7):e0236087.

34. Schroeder‐Kurth T, Schaffert G, Koeckritz W, Kernich M. Quality of life of adults with trisomy 21 living in mental retardation homes compared with those staying under parental care. American Journal of Medical Genetics. 1990;37(S7):317-21.

35. Scott M, Foley K-R, Bourke J, Leonard H, Girdler S. “I have a good life”: the meaning of well-being from the perspective of young adults with Down syndrome. Disability and Rehabilitation. 2014;36(15):1290-8.

36. Thompson T, Talapatra D, Hazel CE, Coleman J, Cutforth N. Thriving with Down syndrome: A qualitative multiple case study. Journal of Applied Research in Intellectual Disabilities. 2020;33(6):1390-404.

37. Thomson GO, Ward KM, Wishart JG. The transition to adulthood for children with Down's syndrome. Disability & Society. 1995;10(3):325-40.

38. van Heumen L, Schippers A. Quality of life for young adults with intellectual disability following individualised support: Individual and family responses. Journal of Intellectual & Developmental Disability. 2016;41(4):299-310.

39. Villani ER, Vetrano DL, Damiano C, Paola AD, Ulgiati AM, Martin L, et al. Impact of COVID-19-related lockdown on psychosocial, cognitive, and functional well-being in adults with down syndrome. Frontiers in Psychiatry. 2020;11:578686.
